# Role of *miR-146a* in neural stem cell differentiation and neural lineage determination: relevance for neurodevelopmental disorders

**DOI:** 10.1186/s13229-018-0219-3

**Published:** 2018-06-19

**Authors:** Lam Son Nguyen, Julien Fregeac, Christine Bole-Feysot, Nicolas Cagnard, Anand Iyer, Jasper Anink, Eleonora Aronica, Olivier Alibeu, Patrick Nitschke, Laurence Colleaux

**Affiliations:** 1 INSERM UMR 1163, Laboratory of Molecular and pathophysiological bases of cognitive disorders, Imagine Institute, Necker-Enfants Malades Hospital, 24 Boulevard du Montparnasse, 75015 Paris, France; 2 Paris Descartes–Sorbonne Paris Cité University, 12 Rue de l’École de Médecine, 75006 Paris, France; 3 Department of (Neuro) Pathology, Academic Medical Center, University of Amsterdam, 1105 AZ Amsterdam, The Netherlands

**Keywords:** Autism spectrum disorders, microRNA, Human neural stem cell, Transcriptome

## Abstract

**Background:**

MicroRNAs (miRNAs) are small, non-coding RNAs that regulate gene expression at the post-transcriptional level. miRNAs have emerged as important modulators of brain development and neuronal function and are implicated in several neurological diseases. Previous studies found *miR-146a* upregulation is the most common miRNA deregulation event in neurodevelopmental disorders such as autism spectrum disorder (ASD), epilepsy, and intellectual disability (ID). Yet, how *miR-146a* upregulation affects the developing fetal brain remains unclear.

**Methods:**

We analyzed the expression of *miR-146a* in the temporal lobe of ASD children using Taqman assay. To assess the role of *miR-146a* in early brain development, we generated and characterized stably induced H9 human neural stem cell (H9 hNSC) overexpressing *miR-146a* using various cell and molecular biology techniques.

**Results:**

We first showed that *miR-146a* upregulation occurs early during childhood in the ASD brain. In H9 hNSC, *miR-146a* overexpression enhances neurite outgrowth and branching and favors differentiation into neuronal like cells. Expression analyses revealed that 10% of the transcriptome was deregulated and organized into two modules critical for cell cycle control and neuronal differentiation. Twenty known or predicted targets of *miR-146a* were significantly deregulated in the modules, acting as potential drivers. The two modules also display distinct transcription profiles during human brain development, affecting regions relevant for ASD including the neocortex, amygdala, and hippocampus. Cell type analyses indicate markers for pyramidal, and interneurons are highly enriched in the deregulated gene list. Up to 40% of known markers of newly defined neuronal lineages were deregulated, suggesting that *miR-146a* could participate also in the acquisition of neuronal identities.

**Conclusion:**

Our results demonstrate the dynamic roles of *miR-146a* in early neuronal development and provide new insight into the molecular events that link *miR-146a* overexpression to impaired neurodevelopment. This, in turn, may yield new therapeutic targets and strategies.

**Electronic supplementary material:**

The online version of this article (10.1186/s13229-018-0219-3) contains supplementary material, which is available to authorized users.

## Background

Studies now indicate that epigenetic modifications play a role in neurodevelopmental disorders. The heritability rate of autism spectrum disorder (ASD) is over 50% with the remaining attributed to environmental/epigenetic factors [1]. MicroRNA (miRNA), one such factor, fine-tunes gene expression required for the development and function of cells and organs. Previously, our group and others implicated upregulation of *miR-146a* as the most common miRNA deregulation event in ASD [2, 3] and related neurodevelopmental disorders such as epilepsy [4] and intellectual disability (ID) [2]. In ASD, studies reported *miR-146a* upregulation in olfactory mucosal stem cells [2], skin fibroblasts [2], and a lymphoblastoid cell line [5] sampled from living patients and the frontal cortex of adult post-mortem brain samples [6]. In post mortem samples from ASD brains [7], *miR-146a* promoter correlates with an increased level of the active H3K27ac histone mark suggesting that the observed upregulation is due to transcriptional deregulation. In epilepsy, *miR-146a* is upregulated in astrocytes in region proximal to the lesions [4, 8]. Importantly, treatment with either an *anti-miR-146a* [9] or a *miR-146a* mimic [10] can ameliorate the latency, frequency, and duration of induced seizures in a rat model of temporal lobe epilepsy, emphasizing the causality and the reversibility of *miR-146a* effects. Understanding the functions of this miRNA in the brain may thus offer opportunities to develop treatments that are currently not available for neurodevelopmental disorders.

*miR-146a* is independently transcribed and processed and evolutionary conserved to lower vertebrates such as zebrafish and fruit fly. In the mouse brain, it is expressed ubiquitously during embryonic development [2]. In postnatal stages, its expression becomes restricted to neurons in regions important for higher cognitive and social functions including frontal cortex, amygdala, and hippocampus [2]. *miR-146a* is well known as a suppressor of inflammatory response by targeting *TRAF6* and *IRAK1* [11]. Its role in brain development is less well explored. In vitro data demonstrate that *miR-146a* regulates the homeostasis and function of brain cells in a developmental stage and cell type-specific manner. In primary mouse neural stem cell (NSC) cultured in EGF and FGF2, *miR-146a* overexpression promoted neuronal differentiation and cell cycle exit by targeting *Notch1* [12]. In mature primary mouse neurons, its overexpression altered dendritic arborization [2] and induced AMPA receptor endocytosis [13], while transfection with the *anti-miR-146a* reduced the frequency and amplitude of synaptic transmission [13]. In rat primary NSC cultured in N2 and bFGF, overexpression of *miR-146a* promoted astrocyte differentiation by inhibiting *Syt1* and *Nlgn1* expression [14]. In primary mouse astrocyte culture, *miR-146a* overexpression hindered migration [12] and proliferation rate and increased glutamate uptake capacity [2].

Collectively, these studies suggest that *miR-146a* contributes to the maintenance and differentiation of NSC. Yet, how *miR-146a* controls the equilibrium among key genes that promote or inhibit entry into the neurogenic program in human NSC remains unclear. We also do not know how this extrapolates to human brain development. To address these issues, we combined expression analyses in human brain samples and in vitro studies on H9-derived human NSC (H9 hNSC) modified to overexpress *miR-146a*.

## Methods

### Patient information

Freshly frozen brain samples were acquired from the NIH Neurobiobank from donors with ASD diagnosis and normal controls. There was no difference in the distribution of sex, ethnicity, average age, nor post-mortem interval (PMI) between the ASD and the control groups (results not shown). The causes of death vary; more ASD donors died from accidents, and more control donors died from infection and heart failure (see Additional file 1: Table S1).

### Whole exome sequencing technique and analysis

DNA was extracted from frozen brain samples using QIAamp DNA Mini Kit (51304, QIAGEN) with RNAse A treatment (19101, QIAGEN) following the manufacturer’s instruction. WES libraries were prepared from 3 μg of genomic DNA sheared with a Covaris S2 Ultrasonicator. Exome capture was performed as recommended by the manufacturer with the 51 Mb SureSelect Human All Exon kit V5 (Agilent technologies). Sequencing of the WES libraries was carried on a pool of barcoded exome libraries on a HiSeq2500 (Illumina) using the HighOutput mode (48 WES libraries per FlowCell). 76+76 paired-end reads were generated. After demultiplexing, paired-end sequences were mapped on the human genome reference (NCBI build37/hg19 version) using BWA. The depth of coverage obtained for each sample was > 80× with > 90% of the exome covered at least by 15×. SNPs and indels calling were made using GATK tools. An in-house software (PolyWeb) was used to annotate and filter the variants. Variant filtering was performed as previously described. Following a published protocol [2], we performed whole exome sequencing (WES) to identify possible deleterious single nucleotide variants (SNVs) in known ASD genes (as collated in SFARI Database) and intellectual disability genes (Necker ID-Panel) in patients (see Additional file 1: Table S2). SNVs in two patients (5308 and 4721) are known single nucleotide polymorphism (SNPs) and unlikely to be pathogenic. We could not confidently establish a genetic cause for most patients, except one. Patient 1349 carried a heterozygous SNV leading to premature termination codon in *SEMA5A* (OMIM 209850), which could explain his phenotype.

### Extraction and analysis of miRNAs

Approximately 50 mg of frozen brain samples were lysed in 600 μl of Lysis/Binding solution from the miRVana™ miRNA isolation kit (AM1560, ThermoFisher Scientific) using FastPrep Lysing Matrix D (116913500, MP Biomedicals) on a FastPrep®-24 Instrument (MP Biomedicals). The samples were spinned for 30 s at 13,000 RPM to reduce bubbles, after which extraction was performed according to the instruction of the manufacturer to obtain total RNA. The concentration was checked by NanoDrop 2000 (ThermoFisher Scientific). Expression profiles of *miR-146a* and 2 housekeeping miRNAs (*miR-106a* and *miR-17*) were assessed using Taqman assays on Fluidigm 98.98 array in technical quadruplicates. The qPCR analysis was carried out on the qPCR-HD-GPC core facility of the ENS and was supported by grants from Région Ile de France. The analysis was performed using mean of housekeeping miRNAs and average of all controls as references.

### Human neural stem cell culture and differentiation

GIBCO® hNSC (H9 hESC-Derived) was purchased commercially (N7800100, ThermoFisher Scientific). Cells were cultured in flasks or plates previously coated for at least 1H with GelTrex™ LDEV-Free, hESC-qualified, reduced growth factor basement membrane matrix (A1413302, ThermoFisher Scientific). Cells were maintained in Complete StemPro® NSC SFM medium (growth media) consisting of KnockOut™ D-MEM/F-12 (12660012, ThermoFisher Scientific) supplemented with 2% StemPro® Neural Supplement (A1050901, ThermoFisher Scientific), 20 ng/mL EGF (PHG0315, ThermoFisher Scientific), 20 ng/mL bFGF (GF003, ThermoFisher Scientific), 2 mM GlutaMax™-I (35050038, ThermoFisher Scientific), and 10 μg/mL Penicillin-Streptomycin (15,140,122, ThermoFisher Scientific). For passaging, cells were washed once with DPBS (14190169, ThermoFisher Scientific) and detached from the surface by StemPro™ Accutase (A1110501, ThermoFisher Scientific), centrifuged at 1500 RPM for 5 min and replated in fresh growth media. To initiate spontaneous differentiation, H9 cells were plated at a density of 20,000 cells/cm^2^ in growth media for 24 h, after which media was replaced with differentiating media (growth media without growth factors). Differentiating media was changed every 2–3 days during the course of the differentiation process.

### Infection and selection of stably integrated H9 cells

The seed of the *miR-146a-5p* in the pLenti-III-miR-146a-GFP construct (mm10082, ABM) 5′-T(GAGAACTG)AATTCCATGGGTT-3′ was destroyed using the QuikChange site-directed mutagenesis kit (200515, Agilent) to produce to *miR-146a-Mut* construct 5′-T(_ATAGGAG)AATTCCATGGGTT-3′. Lentivirus were then produced from the construct by the Plateforme Vecterus Viraux et Transfert de Gènes from Hospital Necker as a paid service. Viral titer was determined by FACS of GFP signal. Virus infection was performed at the multiplicity of infection (MOI) of 5 in growth media, which was replaced after 24 h. Cells were grown in growth media for 72 h; Puromycin (11113802, ThermoFisher Scientific) at 1 μg/ml was then added to the media for 72 h to eliminate all cells without stable integration of the viral DNA containing the transgene and the Puromycin resistance gene. Cells were recovered in normal growth media until reaching confluence. All experiments were performed at the earliest passages possible.

### Proliferation and apoptosis rate analysis

Cells were plated on coated 96-well plate (92096, TPP) in normal growth media. Twenty-four hours after, media was replaced with growth media containing 1/200 Annexin V Incucyte reagent (4641, Essen Bioscience). Cells were cultured inside the Incucyte® Live Cell Analysis System (Essen Bioscience), and images were taken at determined interval consecutively during several days using × 20 objective and at multiple spots per well. In post-analysis, a mask was designed using an inbuilt apoptosis analysis module to count the red apoptotic cells and the cell confluence percentage, which was used to normalize for cell numbers (see Additional file 2: Figure S2a). The same mask was applied to all time points and all repeats.

### Differentiation analysis

Cells were plated on coated 24-well plate (92024, TPP) in normal growth media. Twenty-four hours after, growth media was replaced with differentiating media, which was refreshed every 2–3 days. Cells were cultured inside the IncuCyte® Live Cell Analysis System (Essen Bioscience), and images were taken every 3 h for 14 days using × 20 objective and at 25 different spots per well. In post analysis, a mask was designed using the inbuilt neurite analysis module to detect neurites and cell body. The same mask was applied to all time points and all repeats to record neurite extension over time.

### FACS analysis

Cells were plated on coated T25 tissue flask (90025, TPP) in normal growth media. Twenty-four hours after, growth media was replaced with differentiating media, which was refreshed every 2–3 days. Cells were collected at 1 and 2 weeks into the differentiation process by scraping the surface of the vessels. Cells were then washed once with DPBS and incubated on ice for 30 min with the blue fluorescent reactive dye from the Live/dead fixable dead cell stain kit (L23105, ThermoFisher Scientific). Cells were washed once with DPBS and fixed in 1 ml of IC Fixation Buffer (FB001, ThermoFisher Scientific) for 5 min followed by 2 washes in IC Permeabilization Buffer (PB001, ThermoFisher Scientific) + 1% BSA. Cells were resuspended into 100 μl of IC Permeabilization Buffer + 1% BSA and primary antibodies at the desired concentrations for 1 h on ice. Cells were washed twice in IC Permeabilization Buffer + 1% BSA and incubated in the same media with the secondary antibodies at desired concentrations for 1 h on ice. Cells were washed twice with DPBS + 1% BSA and analyzed immediately on the BD FACS Aria II machine (BD Biosciences). Primary antibody list: polyclonal rabbit anti-NESTIN (N5413, Sigma), monoclonal mouse anti-TUB-III eFluor® 570 conjugated (41-4510, eBioscience), monoclonal mouse anti-GFAP eFluor® 660 (50-9892, eBioscience); secondary antibody list: donkey anti-rabbit Alexa Fluor 555 (A31572, ThermoFisher Scientific).

### RNA sequencing technique and analysis

We tested cells from three independent experiments using three consecutive passages. Total RNA was extracted using TRizol reagent (15596026, ThermoFisher Scientific) and RNeasy Mini Kit (74104, QIAGEN) with DNase I treatment step (79254, QIAGEN) following the manufacturer’s protocol. The integrity of RNA was determined by RNA ScreenTape (5067-5576, Agilent Technologies) on the Agilent 4200 TapeStation (Agilent Technologies). RNA-seq libraries were prepared starting from 600 ng of total RNA using the TruSeq Stranded mRNA LT Sample Prep Kit (Illumina) as recommended by the manufacturer. Half of the oriented cDNA produced from the poly-A+ fraction were PCR amplified (11 cycles). RNA-seq libraries were sequenced on an Illumina HiSeq2500 (Paired-End sequencing 130 × 130 bases, High Throughput Mode). A minimum of 10 million of paired-end reads was produced per library sample. Sequence reads were aligned to the human HG19 reference genome using the Burrows-Wheeler Alignment version 0.6.2.13. Raw and processed data for all samples are available for download from Gene Expression Omnibus (https://www.ncbi.nlm.nih.gov/geo/) under accession number GSE100670. RNA-Seq data were analyzed using a combination of three different tools namely limma [15], DESeq2 [16], and edgeR [17].

### Quantitative reverse transcription PCR analysis (RT-qPCR)

To validate the RNA-Seq results, we selected 27 genes with relevant neuronal function or corresponding to known *miR-146a* targets and tested their expression by quantitative RT-PCR (RT-qPCR) on the Fluidigm 48.48 chip. As six of these genes were identified as DEGs in both conditions, this analysis included 33 comparisons. The qPCR analysis was performed at the qPCR-HD-GPC core facility of the ENS and was supported by grants from Région Ile de France. The analysis was performed using geometric mean of four housekeeping genes (*CYC1*, *GPBP1*, *RPL13A*, and *SDHA*) and average of the *miR-146a-Mut* repeats as references. No significant difference was detected for the 4 housekeeping genes. Lists of primers can be found in Additional file 1: Table S5.

### In silico analyses

Known targets of *miR-146a* were extracted from miRTarBase [18]. Predicted targets were extracted from miRDIP [19]; only genes predicted by at least three different programs were considered. Target prediction of miR-146a-mut was performed using miRDB [20]. Pathway and function enrichment was analyzed using Ingenuity Pathway Analysis (QIAGEN). Protein-protein interaction map was extracted from STRING database [21] and further analyzed using Cytoscape V3.4.0 (http://www.cytoscape.org). Protein interaction enrichment was identified by ClusterOne Plugin downloaded from Cytoscape database. Analyses of expression in different brain regions throughout lifespan and brain cell type enrichment were performed using published code [22] in R program; please refer to the original publication for detailed description. Graphs and statistical analyses were generated by Partek Genomics Suite V6.0.

### Dual luciferase assay

The 3′ untranslated region (UTR) of *DCX*, *GAD1*, and *PAK3* were subcloned into the psiCheck2 plasmid (Promega). Due to their size, the 3′UTR of *DCX* and *PAK3* were cloned into 2 (924 and 810 bp) and 3 parts (1106, 673, and 524 bp), respectively; each of these parts contains at least 3 predicted *miR-146a* binding sites. For luciferase assay, 2 × 10^5^ HEK293T cells were plated in each well of a 6-well plate the day before transfection. Cells were transfected with the psiCheck2 3′UTR clones and the synthetic *miR-146a* (4464067, ThermoFisher Scientific) or *miR-mimic* for control (4464058, ThermoFisher Scientific) using JetPRIME® Polyplus Transfection Reagent (Ozyme) following the manufacturer’s instruction. Assay were performed using the Dual-Luciferase® Reporter Assay System (Promega) 48 h after transfection. Ratio of Renilla to Firefly luciferase was taken as mean of technical triplicates. Three independent transfections were performed to confirm the results.

## Results

### *miR-146a* overexpression occurs early during human brain development

**Fig. 1:**
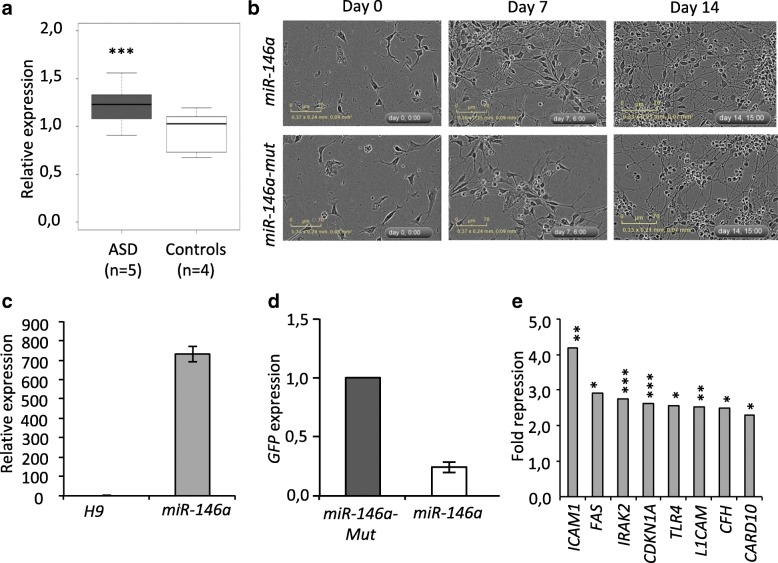
Generation and characterization of H9 hNSC overexpressing *miR-146a*. **a** Expression of *miR-146a* in the brain of ASD patients. Expression was measured in the temporal lobe of ASD patients (white box) and age-matched controls (gray box). Box plot showing relative expression of *miR-146a* measured by Taqman RT-qPCR for two house keeping miRNAs (*miR-106a* and *miR-17*) and the average of all controls. ***Fold change > 1.2 and *P* < 0.001 by Mann Whitney Test. **b** Phase contrast image showing morphology of hNSC lines in undifferentiated (day 0) and differentiated condition upon withdrawal of GF (days 7 and 14). **c** Expression of *miR-146a* (±S.D.) in H9 hNSC with stable construct compared to untransduced cells. Analyses were measured by Taqman assay, normalized against U6. **d** Expression of GFP transgene (±S.D.) in the two newly established hNSC H9 overexpressing *miR-146a* or *miR-146a-Mut*. Relative expression was measured by RT-qPCR, normalized against *GPBP1*. **e** Repression ratio (*miR-146a/miR-146a mut*) of known targets of *miR-146a* in undifferentiated H9 hNSC by RNA-Seq analyses. *P < 0.05, **P < 0.01, ***P > 0.001 by EdgeR analysis

ASD, which is diagnosed before the age of 3, is associated with brain defects arising during early development, including enlarged brain volume (2–5%) that ameliorates in later stages [23] and disorganized cortical layers [24]. As a first step to understand how *miR-146a* overexpression may contribute to ASD, we asked whether it could be detected early during brain development. Using a Taqman assay, we analyzed the expression of *miR-146a* and two housekeeping miRNAs (*miR-106a* and *miR-17*) in the temporal lobe (Brodmann’s Area 21) of ASD children (4–9 years old) (see the “Methods” section and Additional file 1: Tables S1, S2 for detailed sample description). We observed a 1.3-fold increase in *miR-146a* expression in the patient samples compared to controls (see Fig. 1a), demonstrating that *miR-146a* upregulation is observed during the early stages of ASD progression/emergence.

### Generation and validation H9 hNSC lines overexpressing *miR-146a*

To elucidate the role of *miR-146a* in neural development*,* we used integrating viral approach to establish two H9 hNSC lines stably overexpressing a wild-type *miR-146a* gene or its mutant form, *miR-146a-Mut* (see Fig. 1b). Transgene expression was validated by either Taqman assay for *miR-146a* (see Fig. 1c) or measurement of the GFP transcript level by RT-qPCR for the *miR-146a-Mut cell* (see Fig. 1d). These lines were also validated using RNA-Seq, which showed a significant downregulation of known *miR-146a* target transcripts [18], including *ICAM1*, *FAS*, *IRAK2*, *CFH*, *CDKN1A*, *TLR4*, *L1CAM*, and *CARD10* (see Fig. 1e). Predicted targets of *miR-146a* were more frequently downregulated compared to those of *miR-146a-Mut* (20 vs. 5 targets with fold change > 1.5 and *P* < 0.05 in undifferentiated cells; *P* < 0.01 by Fisher’s Exact Test), suggesting that off-target effect due to *miR-146a-Mut* overexpression was minimal. In addition, array comparative genomic hybridization analysis did not identify any copy number variation, indicating that the genomes of the newly established lines were stable (results not shown).

### *miR-146a* promotes hNSC differentiation

**Fig. 2:**
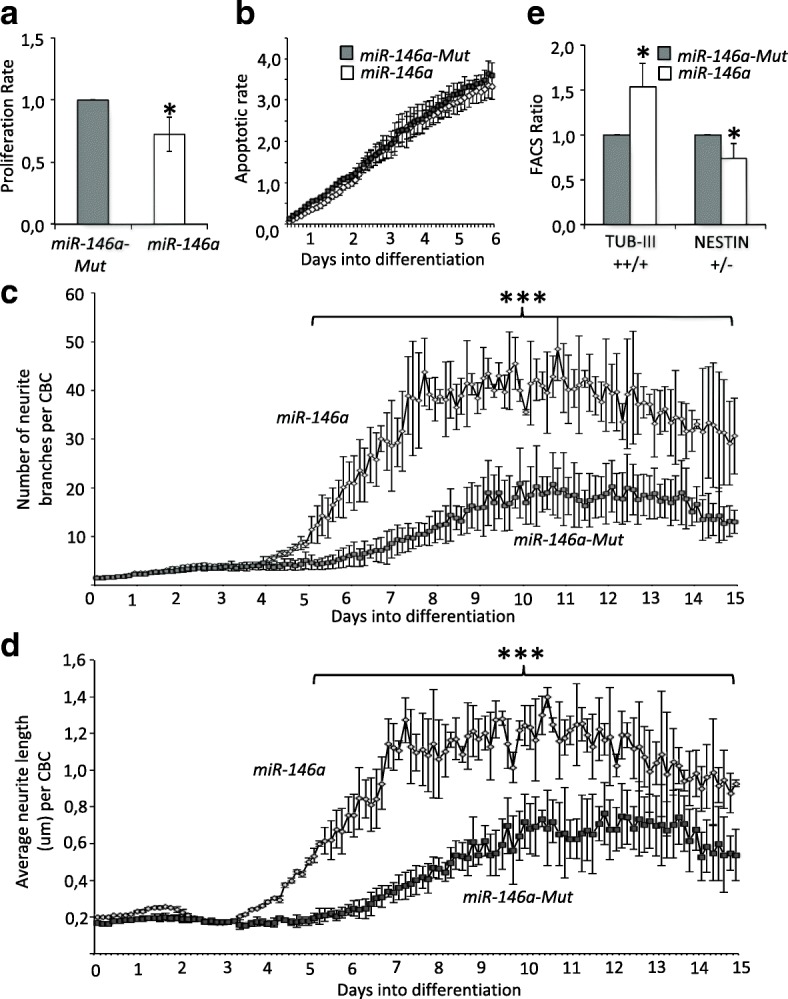
*miR-146a* controls responses of H9 hNSC to neurogenic cues. **a** Cell proliferation rates measured using the Incucyte machine over 48 h after induction of differentiation. Graph shows the average ratio (±S.D.) of proliferation slopes from 3 independent experiments. *P < 0.05 by Student’s 2-tail unpaired *T* test. **b** Apoptotic cell rates measured by the number of Annexin V labeled cells over the confluence percentage using the Incucyte machine. Graph shows average ratio (±S.D.) of four technical replicates and are representative of two independent repeats showing the same results. **c** Average number of neurite branching (±S.D.) and average neurite length **d** (±S.D.) in cells undergoing differentiation over 2 weeks. Analyses were performed using an analysis mask (see Additional file 2: Fig. S1a) on images taken at 25 different spots every 3 h over 15 days by the Incucyte machine. Results are representative of three independent repeats showing similar results. ***P < 0.001 by Student’s 2-tailed unpaired *T* test. **e** FACS analysis was performed on differentiated cells labeled by TUB-III or NESTIN antibodies (see Additional file 2: Figure S2 for details). Graph shows average ratio (±S.D.) of three independent repeats. *P < 0.05 by Student’s 2-tail unpaired *T* test

We then assessed the effects of *miR-146a* overexpression using live cell imaging (see Additional file 2: Figure S1a). In normal growth conditions, overexpression of *miR-146a* had no effect on the proliferation rate or the apoptotic rate of the cells (see Additional file 2: Figures S1b ,c). In contrast, upon induction of differentiation by withdrawal of EGF and bFGF growth factors, we observed a significant decrease in the proliferation rate (see Fig. 2a). The apoptotic rates of the *miR-146a* and *miR-146a-Mut* lines increased during cell differentiation, but there was no difference between them (see Fig. 2b). Importantly, over the 2-week course of differentiation, we observed increased dendritic branching (see Fig. 2c) and extension (see Fig. 2d) in the *miR-146a* line compared to control. Undifferentiated H9 hNSC co-express NESTIN, β-III-TUBULIN (TUB-III), and GFAP [25], which are markers for progenitor, differentiated neuron and astrocyte, respectively. As differentiation proceeded, some cells lost their NESTIN expression and increased TUB-III expression, while GFAP level remained unchanged (see Additional file 2: Figure S2a,b). We calculated the ratio of cells expressing high level/low level of TUB-III and the number of NESTIN positive/negative cells after differentiation. We found that *miR-146a* overexpression induces enhanced level of TUB-III and reduced levels of NESTIN (see Fig. 2e). Collectively, these results suggest that *miR-146a* overexpression favors differentiation of H9 hNSC into neurons in response to neurogenic cue.

### *miR-146a* overexpression alters the balance between neural progenitor cell renewal and neuronal differentiation

**Fig. 3:**
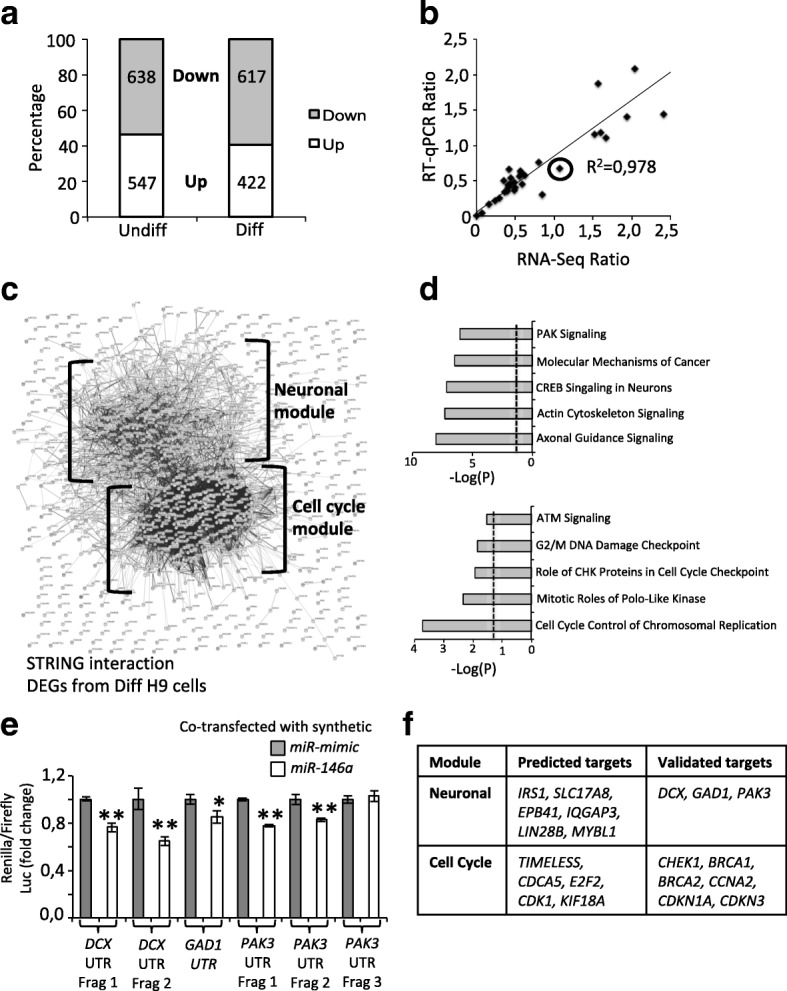
*miR-146a* targets key genes to promote hNSC differentiation. **a** Distribution of DEGs identified by RNA-Seq in undifferentiated (Undiff) and differentiated (Diff) conditions. **b** Graph shows expression ratios of DEGs measured by RNA-Seq and RT-qPCR. Circle indicates only incompatible/non-validated DEGs. **c** Protein interaction network of all DEGs identified in differentiated cells mapped by STRING. Two modules are identified: Neuronal Module (top) contains 350 genes and Cell Cycle Module (bottom) contains 155 genes. **d** Top 5 pathways identified by Ingenuity Pathway Analysis of the 2 modules (see Additional file 1: Tables S8, S9 for extended lists). **e** *miR-146a* directly targets *DCX*, *GAD1*, and *PAK3* 3′UTR. Due to their size, the 3′UTR of *DCX* and *PAK3* were cloned into 2 and 3 fragments (frag), respectively. Each 3′UTR construct contains at least 3 predicted *miR-146a* binding sites. Ratio of Renilla/Firefly luciferase (±SD of 3 technical triplicates) was measured in cell lines co-transfected with either the synthetic *miR-mimic* (control) or the *miR-146a*. Graphs are representative of 3 independent assays showing the same results. *P < 0.05, **P < 0.01 by Student’s 2 tailed paired *t* test. **f** List of known and predicted *miR-146a* targets that are downregulated in each respective module

miRNAs regulate multiple signaling pathways through their interaction with hundreds of different transcripts. To identify the mechanisms that could contribute to neural differentiation in *miR-146a* overexpressing hNSC, we performed expression analyses. Proteomic analysis is a valuable approach to identify changes in protein levels regulated by *miR-146a*, yet, it presents important limitations including limited access to low abundant proteins which expression is triggered during differentiation. Thus, we decided to perform RNA-Seq analyses in both undifferentiated and differentiated cells to identify differentially expressed genes (DEGs) and corresponding affected pathways that may mediate the effect of *miR-146a* overexpression on neural differentiation. Compared to *miR-146a-Mut* using a threshold of *P* < 0.05 and fold change > 1.5, we detected 1185 DEGs (10% of total detected transcripts, 54% downregulated, and 46% upregulated) in undifferentiated cells (see Fig. 3a and Additional file 1: Table S3) and 1039 DEGs (9% of total detected transcripts, 59% down, and 41% upregulated) in differentiated cells (see Fig. 3a and Additional file 1: Table S4). We obtained a 97% validation rate by RT-qPCR (32/33 genes validated, *R*^2^ = 0.978; see the “Methods” section; see Fig. 3b and Additional file 1: Table S5). We further validated the downregulation of PAK3 in undifferentiated and differentiated H9 NSC, and the upregulation of NOTCH1 in differentiated cells by western blot (Additional file 2: Figure S3).

Next, we sought to identify the deregulated pathways. We first extracted high confident known and predicted interactions from STRING database [21]. In undifferentiated cells, pathway analyses suggested that DEGs affect very different classes of biological processes including axonal guidance, colorectal cancer, germ Cell-Sertoli Cell Junction Signaling **(**see Additional file 1: Table S6). Moreover, gene/protein interacting network was random and did not organize into functional modules (see Additional file 2: Figure S4). In contrast, analysis on data obtained from differentiated cells revealed enrichment for two networks (see Fig. 3c). Using Cytoscape with ClusterOne plugin, we found that the first network consisted of 155 genes comprising of four modules (see Additional file 2: Figure S5 and Additional file 1: Table S7**)**. Remarkably, all genes from this network except one are downregulated (154/155 DEGs, *P* = 0 by Fisher’s Exact Test) and were enriched for cell cycle control pathways (Cell Cycle Module; see Fig. 3d and Additional file 1: Table S8). We further analyzed the remaining 884 genes by filtering out those encoding proteins with no or only one interaction (those in the outer edge). This created a second list of 350 genes with an equal distribution of up or downregulated DEGs (195 down, 153 upregulated, *P* > 0.05 by Fisher’s Exact Test). Importantly, a significant enrichment for pathways related to neuronal differentiation was observed in this dataset (Neuronal Module; see Fig. 3c and Additional file 1: Table S9). To identify the drivers that could mobilize the observed interactions, we search for known and predicted *miR-146a* targets (as collated in miRTarBase [18] and using at least 3 different programs as collated by miRDiP [19]) that co-localized to this network (see Additional file 2: Figure S6). We chose to test the interaction between *miR-146a* and *PAK3*, *DCX* and *GAD1* by luciferase assays for their crucial roles in NSC differentiation and neuronal migration. This analysis revealed that *miR-146a* directly targets the 3′UTR of all three genes (see Fig. 3e). Taken together, our results suggested that *miR-146a* promotes cell cycle exit and neuronal differentiation by targeting 11 key cell cycle genes (*CHEK1*, *BRCA1*, *BRCA2*, *CCNA2*, *CDKN1A*, *CDKN3*, *TIMELESS*, *CDK1*, *CDCA5*, *E2F2* & *KIF18A*) and 9 neuronal genes (*DCX*, *PAK3*, *IRS1*, *GAD1*, *SLC17A8*, *EPB41*, *MYBL1*, *IQGAP3* & *LIN28B*), 9 of which are validated targets while 11 are predicted targets of *miR-146a* (see Fig. 3f).

### *miR-146a* overexpression affects specific regions in early human brain development

**Fig. 4:**
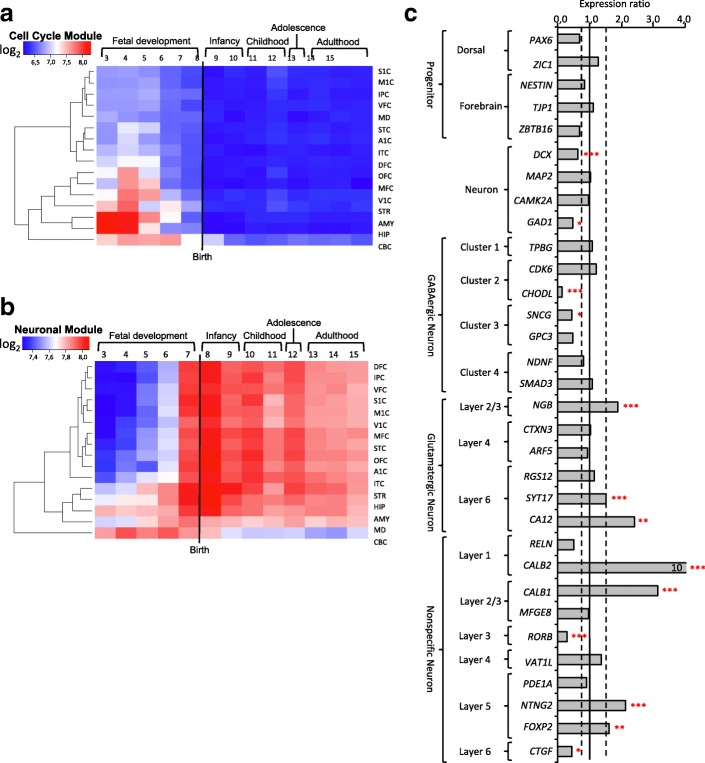
*miR-146a* determines neuronal lineage identities. Heatmap of gradient of expression from the Cell Cycle (**a**) and Neuronal (**b**) Modules spanning human fetal development to late adulthood in distinct brain regions. A1C, auditory cortex; AMY, amygdala; CBC, cerebellar cortex; DFC, dorsolateral prefrontal cortex; HIP, hippocampus; IPC, posterior inferior parietal cortex; ITC, inferior temporal cortex; M1C, primary motor cortex; MD, mediodorsal nucleus of thalamus; MFC, medial prefrontal cortex; OFC, orbital prefrontal cortex; S1C, primary somatosensory cortex; STC, superior temporal cortex; STR, striatum; V1C, primary visual cortex; VFC, ventrolateral prefrontal cortex. Period labels: 3—Early fetal (10 ≤ Age ≤ 13 Post conception week, PCW); 4—Early midfetal (13 ≤ Age ≤ 16 PCW); 5—Early midfetal (16 ≤ Age ≤ 19 PCW); 6—Late midfetal (19 ≤ Age ≤ 24 PCW); 7—Late fetal (24 ≤ Age ≤ 38 PCW); 8—Neonatal and early infancy (Birth ≤ Age ≤ 6 Postnatal months, M); 9—Late infancy (6 ≤ Age ≤ 12 M); 10—Early childhood (1 ≤ Age ≤ 6 Postnatal years, Y); 11—Middle and late childhood (6 ≤ Age ≤ 12 Y); 12—Adolescence (12 ≤ Age ≤ 20 Y); 13—Young adulthood (20 ≤ Age ≤ 40 Y); 14—Middle adulthood (40 ≤ Age ≤ 60 Y); 15—Late adulthood (60 Y ≤ Age). **c** Expression difference of detectable neuronal specific markers in differentiated H9 hNSC. Dotted lines indicate expression difference threshold (fold change ≥ 1.5). *P < 0.05, **P < 0.01 and ***P < 0.001 by EdgeR analysis (as representative of 3 different RNA-Seq analyses)

To identify the developmental stages and regions of the human brain potentially affected by the overexpression of *miR-146a*, we investigated the expression of the DEGs in a comprehensive dataset on the human brain transcriptome previously described [22, 26]. We found that expression of genes in the Cell Cycle Module is specifically restricted to early stages of fetal development in the hippocampus, amygdala, visual cortex, medial prefrontal cortex, and cerebellum (see Fig. 4a). By contrast, we observed a developmental gradient of expression of genes from the Neuronal Module that increases during fetal development in the hippocampus and amygdala, peaks at an early infancy stage in all regions of the brain and persists throughout the life. Cerebellar expression seems restricted to prenatal stages (see Fig. 4b). Notably, this Neuronal Module, but not Cell Cycle Module, is significantly enriched for ASD-associated genes (*P* < 0.001 by Fisher’s Exact Test, see Additional file 1: Table S4).

To further explore the contribution of *miR-146a* upregulation on the transcriptome of ASD brain, we compared the DEGs identified in differentiated H9 hNSC to the published DEGs identified in ASD adult post-mortem brains (cortex, temporal lobe, and frontal cortex) [27]. We identified 42 overlapping genes that share the same trend of deregulation between the two studies (6.3% of published DEGs detectable in H9 hNSC) (see Additional file 1: Table S4). Half of these genes (20/42) belong to the Neuronal Module, whereas only two belong to the Cell Cycle Module. This analysis suggested that upregulation of *miR-146a* could be responsible for a portion of transcriptomic changes seen in ASD brains and that the use of H9 hNSC could provide relevant insights into both early processes of neural differentiation and later stages of neuronal development.

### *miR-146a* overexpression correlates with abnormal lineage-specific gene expression

The central nervous system contains many diverse neuronal subtypes that have been classified into 50 different groups based on single-cell gene expression profiles [28]. Since *miR-146a* overexpression alters hNSC differentiation, we asked whether it also affects neural cell fate programming and impacts neuronal specialization. Our RNA-seq data indicated that the Neuronal Module is enriched for genes expressed in pyramidal neurons and interneurons (*P* < 0.05; see Additional file 1: Table S10) [22], even though the Cell Cycle Module is broadly expressed in all cell types (see Additional file 1: Table S10). When examining the expression of all 87 established cortical layer-specific, neuronal-specific, and progenitor-specific markers from published sources [24, 28, 29], we observed that 40% (13/32) of detectable markers for differentiated H9 cells were significantly deregulated in hNSC overexpressing *miR-146a* (see Fig. 4c). These markers were for GABAergic neurons (*CHODL*, *GAD1*, *SNCG*), glutamatergic neurons (*NGB*, *SYT17*, *CA12*), layers 2/3 (*CALB1*, *CALB2*), layer 4 (*RORB*), layers 5/6 (*NTNG2*, *FOXP2*, *CTGF*), and migrating neurons (*DCX*) (see Fig. 4c). These results strongly suggest that regulation of *miR-146a* expression level is important to correctly acquire neuronal lineage identities.

## Discussion

In this study, we provided insight into the role of *miR-146a* in brain development and its relevance for neurodevelopmental disorders by combining the analyses of post-mortem human brain samples and in vitro models. We demonstrated that *miR-146a* overexpression in the brain of ASD patient is an early event detectable from childhood at an age when ASD are typically diagnosed (see Fig. 1a). Albeit the limited number of samples tested, this is the fifth time that this miRNA has been found upregulated in an independent cohort [2, 3, 5, 6]. In neural-relevant cell types, upregulation of *miR-146a* has been reported in adult cortex BA10 [6] and in adult olfactory mucosal stem cells (OMSC) [2]. These results collectively suggest that *miR-146a* upregulation is an event that occurs during embryogenesis and continues throughout development.

Our in vitro analyses on hNSC suggest that *miR-146a* contributes to the regulation of balancing cell-cycle exit/cell-cycle re-entry of neural progenitors and committing to neural differentiation pathways. These results are in agreement with previously published studies. Indeed, overexpression of *miR-146a* has been shown to induce cell cycle arrest in normal [2] or malignant mouse astrocytes [12], in human non-small cell lung cancer cells [30] and in mouse NSC [12]. Increased *miR-146a* level also enhanced neuronal differentiation in mouse NSC through suppression of *Notch1* [12]. However, in our study, we found that NOTCH1 was upregulated in differentiated H9 NSC. This can be explained by two major differences: (i) unlike the mouse *Notch1*, the human *NOTCH1* gene is not predicted to be a target of *miR-146a*, and (ii) activation and not suppression of NOTCH1 is required for human NSC differentiation [31]. This emphasizes the relevance of using human H9 NSC to model early neuronal development of patients with ASD. In addition, we propose that *miR-146a* modulates the homeostasis of NSC by targeting directly and concurrently at least 20 different key neuronal and cell cycle genes, 9 of which are validated targets while 11 are predicted targets (see Fig. 3f). Thus, while inhibition of vesicular glutamate transporters (VGLUTs) promotes neuronal differentiation and migration of NPCs [32], we observed that *miR-146a* overexpression caused downregulation of the vesicular glutamate transporter gene *SLC17A8*. Similarly, RNA-Seq data revealed downregulation of the *LIN28B* gene; the RNA binding protein LIN28B plays essential functions in neuroblast proliferation by maintaining neural progenitors in an early state [33]. Lastly, *miR-146a* overexpression downregulates several CDK genes including *CDKN1A*, *CDKN3*, and *CDK1*, which encode for key proteins controlling the length of G_1_ phase during cell-cycle and the balance between progenitor maintenance and generation of differentiated neurons [34].

Cortical cytoarchitecture relies on the spatiotemporal coordination of neuronal production rate, precursors cell-cycle control, and neuronal radial migration toward the cortical plate. Radial glial cells (RGC), the key progenitor cells in the developing CNS, divide asymmetrically to generate a new RGC as well as a post-mitotic neuron or an intermediate progenitor daughter cell. Neurons migrate toward the cortical plate along the fibers of RGC to reach their final position within the nascent neocortex and acquire their specific identity. Migration and final laminar positioning of neurons relies the fine-tuning of cell type- and layer-specific transcription factors [35]. Our results suggest that upregulation of *miR-146a* could disturb these transcriptional programs and may contribute to the disorganization of cortical layers [24] and the increase in number of neurons [36] and dendritic spine density [37] observed in ASD brains.

Data from RNA-Seq indicate that DEGs in H9 hNSC are significantly enriched for markers for pyramidal and interneurons, as well as markers for GABAergic (up regulated) and glutamatergic neurons (down regulated) (see Fig. 4c and Additional file 1: Table S10), suggesting that *miR-146a* may also contribute to the adequate distribution of these neurons. In mouse, artificially enhanced number of pyramidal neurons in the upper neocortical layers impaired neurite extension and laminar distribution of interneurons, ultimately leading to autism-like phenotype [38]. In human, Wegiel et al. examined post-mortem brains of 14 subjects with ASD and reported localized deficit of pyramidal neurons in the CA1 sector in 3 subjects and thickening of pyramidal layer in the CA1 sector in another [39]. The number of PVALB positive interneurons, a GABAergic subtype, was also found significantly reduced in prefrontal cortex BA46, BA47, and BA9 in 11 ASD cases [40]. As such, our results will direct future investigations into the role of *miR-146a* in the signaling cascade mediating the determination and acquisition of neuronal lineage identities.

## Conclusion

The accurate generation of an appropriate number of different neuronal and glial subtypes is fundamental for normal brain functions. It requires tightly orchestrated, spatial, and temporal developmental programs to maintain the balance between neural progenitor cell proliferation and differentiation. While ASD is often considered as caused by synaptic dysfunction [41], several evidence from human neuropathology, systems biology, and developmental biology implicated dysregulation of the cell cycle and cortical lamination in the developing brain as a potential common pathophysiological mechanism underlying ASD [42–45]. Based on our results, we speculate that *miR-146a* plays a dynamic role to shape brain development from early neurogenesis to synaptic maturation and propose that *miR-146a* overexpression could provide a potential unifying explanation for brain dysfunctions observed in neurodevelopmental disorders.

## Additional file

Additional file 1: **Table S1.** Detailed information of all patients and controls whose brain samples were used in this study. **Table S2.** Possibly deleterious variants in known ASD and ID genes. **Table S3.** DEGs identified in undifferentiated cells. **Table S4.** DEGs identified in differentiated cells. **Table S5.** Validation of RNA-Seq using RT-qPCR on Fluidigm array. **Table S6.** Top 20 cannonical pathways deregulated in undifferentiated cells. **Table S7.** Top 4 nodes enriched for protein-protein interaction as calculated by ClusterOne Plugin. **Table S8.** Top 20 cannonical pathways deregulated in the cell cycle modules of differentiated cells. **Table S9.** Top 20 cannonical pathways deregulated in the cell neuronal modules of differentiated cells. **Table S10.** Cell type enrichment analysis of DEGs from the Cell Cycle and Neuronal Modules. (XLSX 888 kb)

Additional file 2: **Figure S1.** Characteristics of undifferentiated H9 hNSC. **Figure S2.** FACS analyses of cell type specific markers NESTIN, GFAP and TUB-III in undifferentiated and differentiated conditions. **Figure S3.** Western blot validation of PAK3 and NOTCH1 expression in undifferentiated and differentiated H9 NSC. **Figure S4.** Protein interaction network of all DEGs in undifferentiated cells predicted by STRING. **Figure S5.** Top four interacting networks corresponding to the cell cycle module in differentiated cells. **Figure S6.** Co-localization of known and predicted targets of miR-146a in the protein interaction network of DEGs in differentiated cells. (PPTX 7099 kb)

## Abbreviations

ASD: Autism spectrum disorder
CNS: Central nervous system
DEG: Differentially expressed gene
hNSC: Human neural stem cell
ID: Intellectual disability
miRNA: MicroRNA
MOI: Multiplicity of infection
OMSC: Adult olfactory mucosal stem cell
PMI: Post-mortem interval
RGC: Radial glial cell
RT-qPCR: Quantitative reverse transcription polymerase chain reaction
SNP: Single nucleotide polymorphism
SNV: Single nucleotide variant
TUB-III: β-III-TUBULIN
WES: Whole exome sequencing
