# Supplementary material for: Role of miR-146a in neural stem cell differentiation and neural lineage determination: relevance for neurodevelopmental disorders
Source: Mol Autism. 2018 Jun 19;9:38. doi: 10.1186/s13229-018-0219-3 (PMC6011198; doi:10.1186/s13229-018-0219-3)
Supplement: Supplementary file 2 — Figure S1. Characteristics of undifferentiated H9 hNSC. Figure S2. FACS analyses of cell type specific markers NESTIN, GFAP and TUB-III in undifferentiated and differentiated conditions. Figure S3. Western blot validation of PAK3 and NOTCH1 expression in undifferentiated and differentiated H9 NSC. Figure S4. Protein interaction network of all DEGs in undifferentiated cells predicted by STRING. Figure S5. Top four interacting networks corresponding to the cell cycle module in differentiated cells. Figure S6. Co-localization of known and predicted targets of miR-146a in the protein interaction network of DEGs in differentiated cells. (PPTX 7099 kb) [file 13229_2018_219_MOESM2_ESM.pptx]

## Slide 1
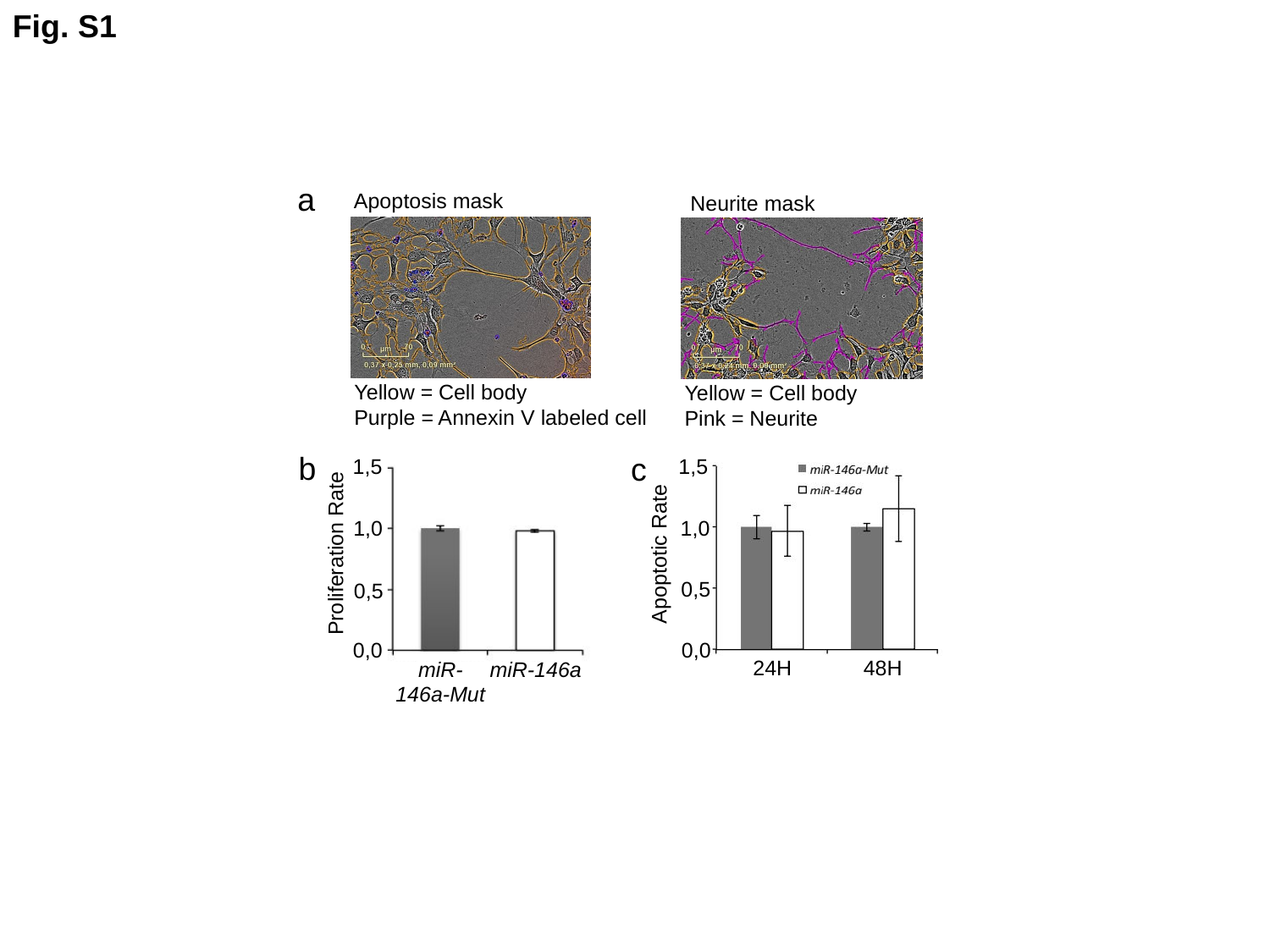

Fig. S1
a
Apoptosis mask
Neurite mask
Yellow = Cell body
Purple = Annexin V labeled cell
Yellow = Cell body
Pink = Neurite
b
1,5
1,0
Proliferation Rate
0,5
0,0
miR-146a-Mut
miR-146a
c
1,5
1,0
Apoptotic Rate
0,5
0,0
24H
48H

## Slide 2
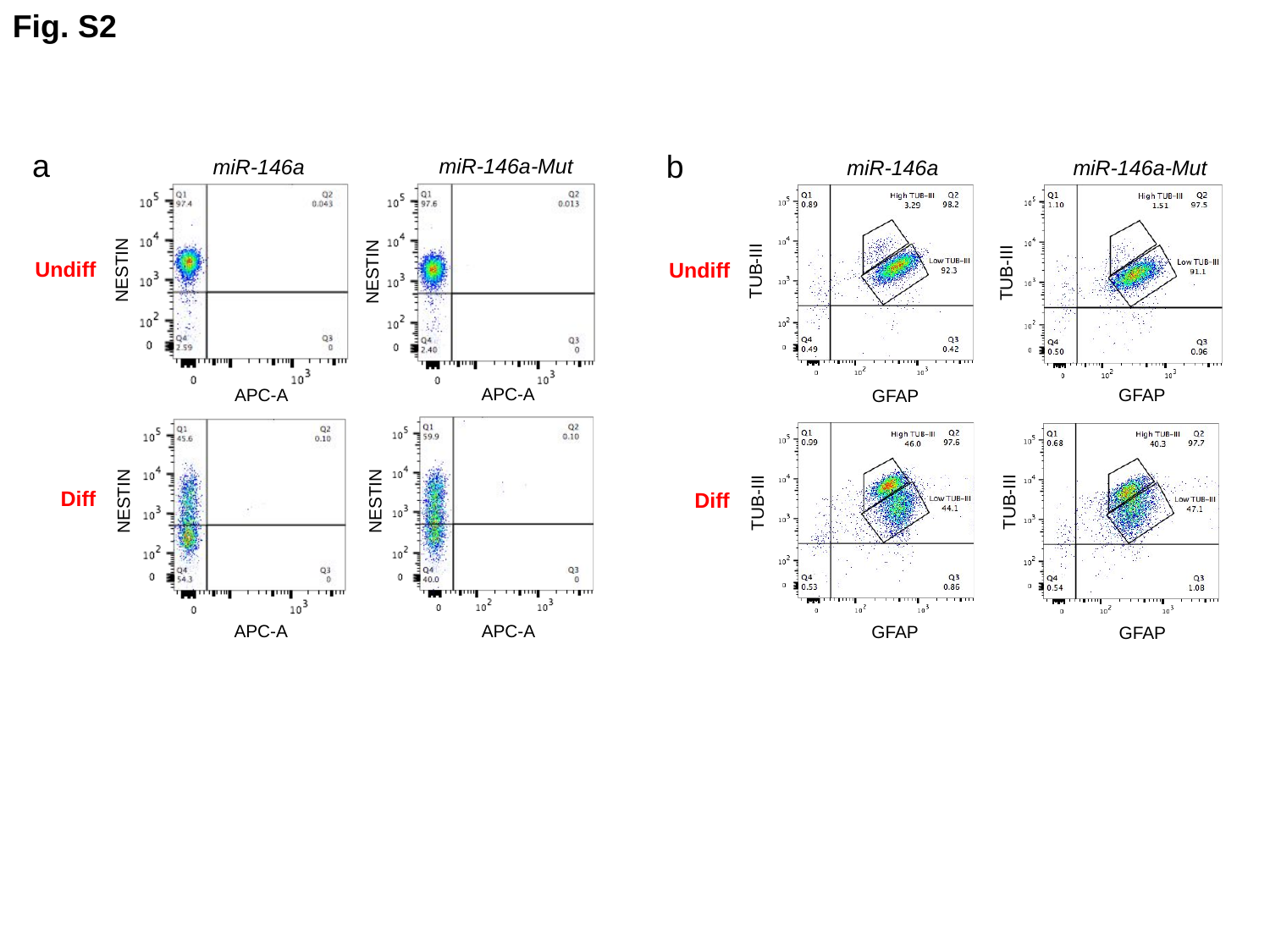

Fig. S2
a
b
miR-146a-Mut
miR-146a
miR-146a-Mut
miR-146a
Undiff
Undiff
NESTIN
TUB-III
NESTIN
TUB-III
APC-A
APC-A
GFAP
GFAP
Diff
Diff
NESTIN
NESTIN
TUB-III
TUB-III
APC-A
APC-A
GFAP
GFAP

## Slide 3
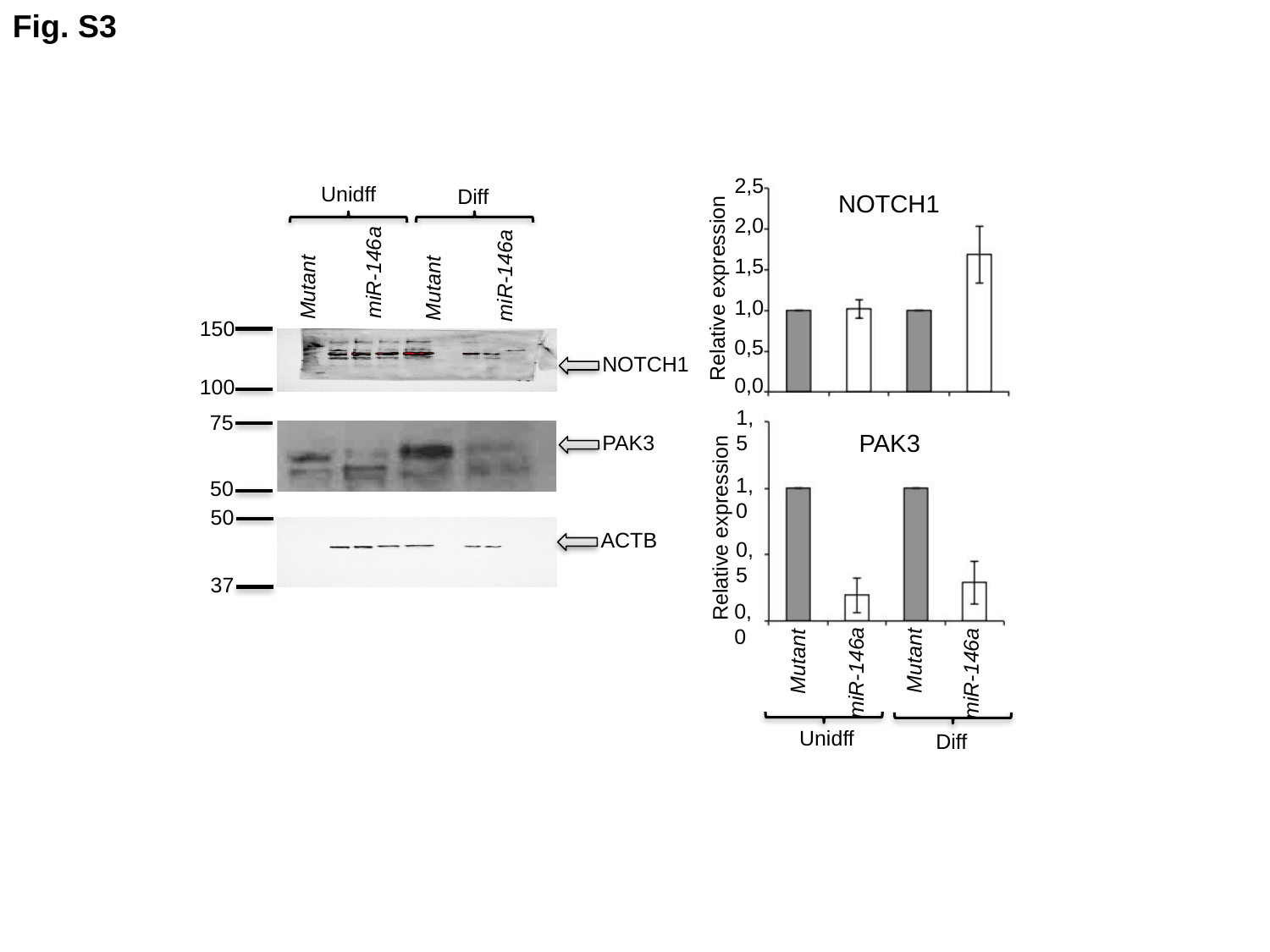

Fig. S3
2,5
2,0
1,5
1,0
0,5
0,0
Unidff
Diff
NOTCH1
miR-146a
miR-146a
Mutant
Mutant
Relative expression
150
NOTCH1
100
1,5
1,0
0,5
0,0
75
PAK3
PAK3
50
50
Relative expression
ACTB
37
Mutant
Mutant
miR-146a
miR-146a
Unidff
Diff

## Slide 4
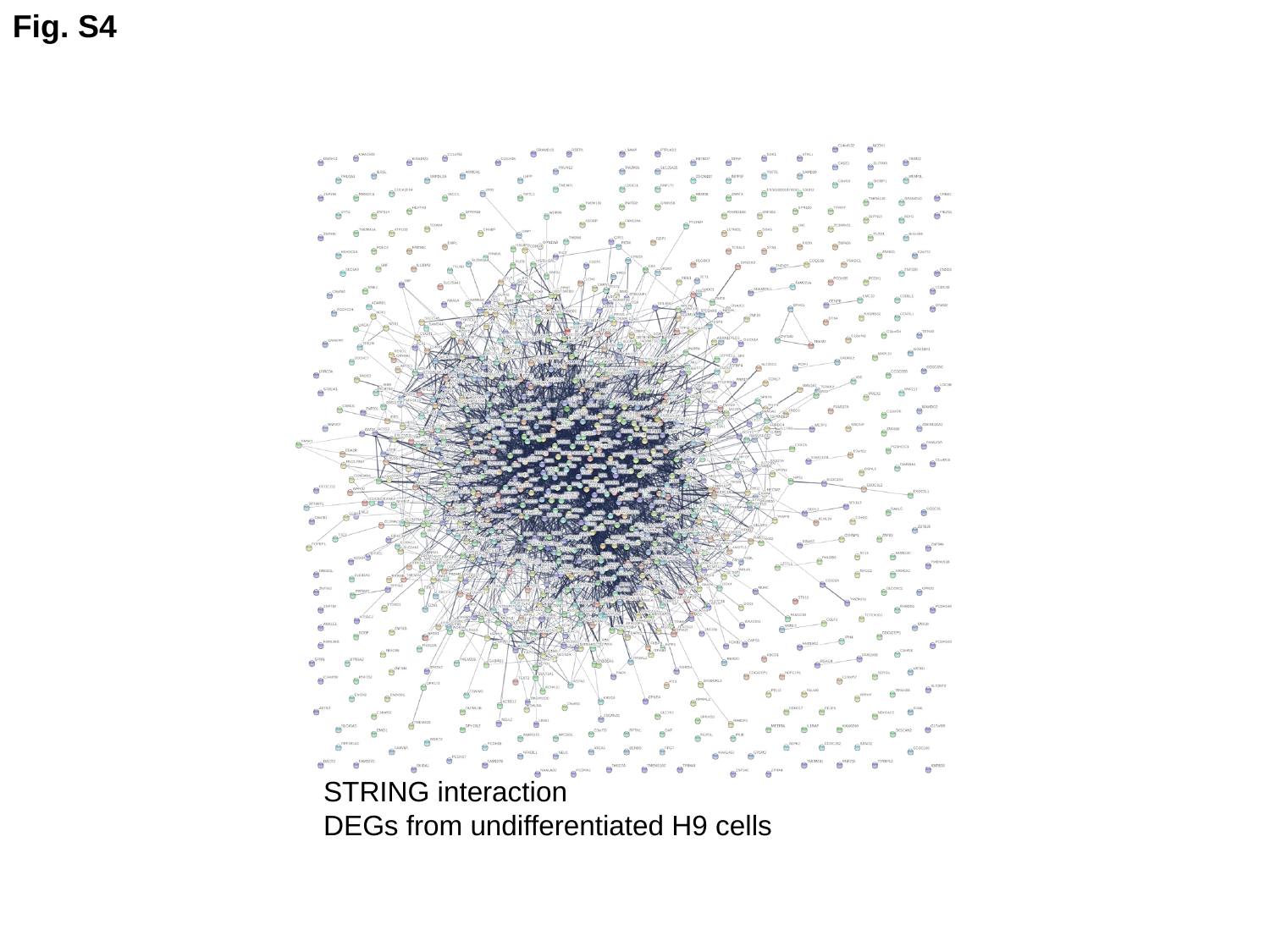

Fig. S4
STRING interaction
DEGs from undifferentiated H9 cells

## Slide 5
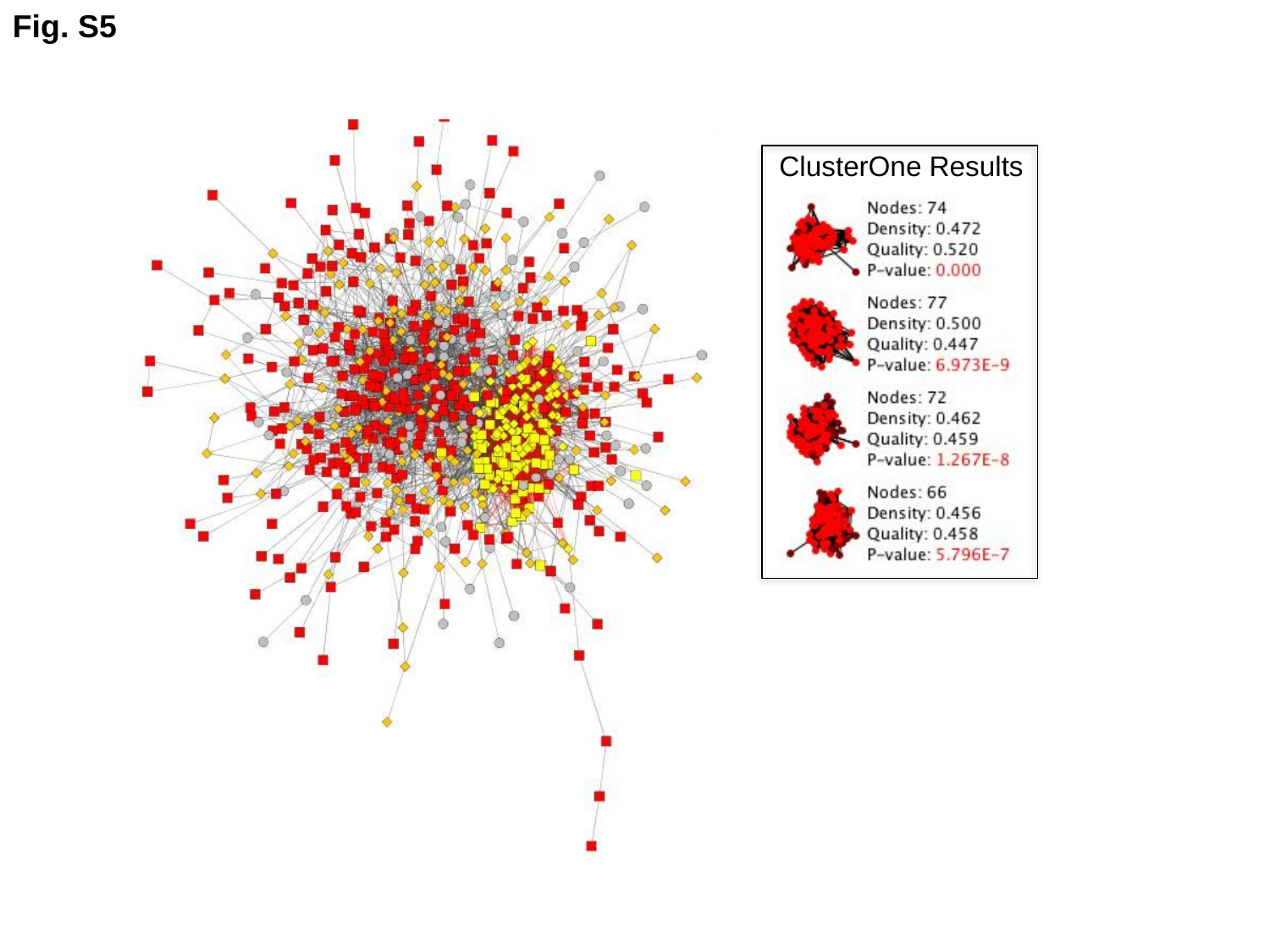

Fig. S5
ClusterOne Results

## Slide 6
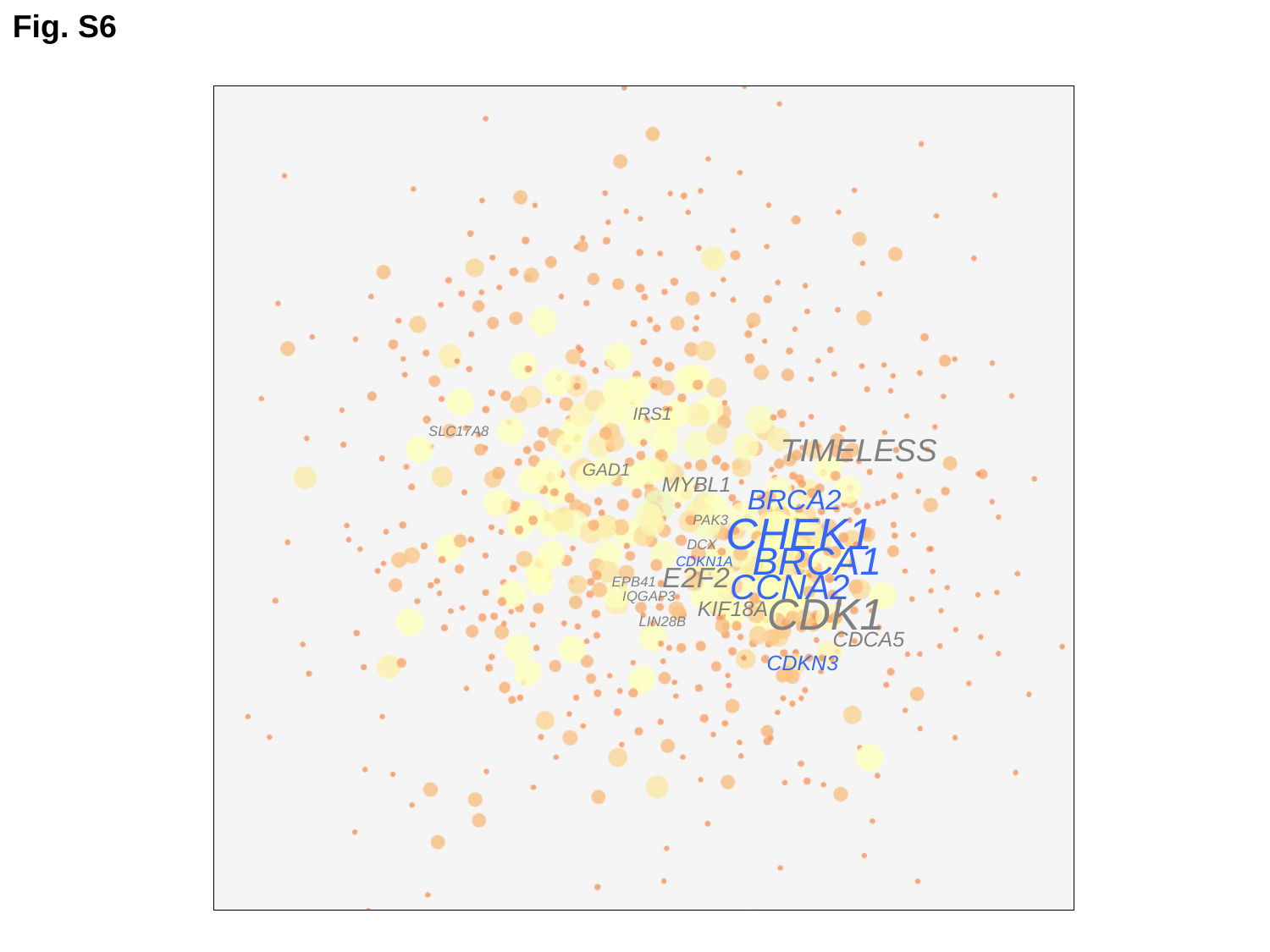

Fig. S6
IRS1
SLC17A8
TIMELESS
GAD1
BRCA2
CHEK1
PAK3
DCX
BRCA1
CDKN1A
E2F2
CCNA2
EPB41
IQGAP3
LIN28B
CDCA5
CDKN3
MYBL1
CDK1
KIF18A
